# Trophic Activity and Phenotype of Adipose Tissue-Derived Mesenchymal Stem Cells as a Background of Their Regenerative Potential

**DOI:** 10.1155/2017/1653254

**Published:** 2017-07-05

**Authors:** Beata Kocan, Aleksandra Maziarz, Jacek Tabarkiewicz, Takahiro Ochiya, Agnieszka Banaś-Ząbczyk

**Affiliations:** ^1^Laboratory of Stem Cells' Biology, Department of Human Immunology, Chair of Preclinical Studies, Institute of Experimental and Clinical Medicine, Faculty of Medicine, University of Rzeszow, Ul. Kopisto 2a, 35-310 Rzeszow, Poland; ^2^Centre for Innovative Research in Medical and Natural Sciences, Faculty of Medicine, University of Rzeszow, Ul. Warzywna 1a, 35-310 Rzeszow, Poland; ^3^Division of Molecular and Cellular Medicine, National Cancer Center Research Institute, Tokyo 104-0045, Japan

## Abstract

There has been an increased interest in mesenchymal stem cells from adipose tissue, due to their abundance and accessibility with no ethical concerns. Their multipotent properties make them appropriate for regenerative clinical applications. It has been shown that adipose-derived stem cells (ASCs) may differ between the origin sites. Moreover, a variety of internal and external factors may affect their biological characteristics, as what we aimed to highlight in this review. It has been demonstrated that ASCs secrete multiple trophic factors that are capable of stimulating cell proliferation and differentiation and migration of various cell types. Particular attention should be given to exosomes, since it is known that they contribute to the paracrine effects of MSCs. Secretion of trophic agents by ASCs is thought to be in a greater importance for regenerative medicine applications, rather than cells engraftment to the site of injury and their differentiation ability. The surface marker profile of ASCs seems to be similar to that of the mesenchymal stem cells from bone marrow, although some molecular differences are observed. Thus, in this review, we have attempted to define trophic activity, as well as phenotypic characterization of ASCs, as crucial factors for therapeutic usage.

## 1. Introduction

Stem cells reside in almost all tissues within the human body where they exhibit various potential. These cells reveal self-renewal capacity, long-term viability, and ability to undergo multiple lineage differentiation in an appropriate microenvironment. They are of great importance for application in regenerative medicine because they control homeostasis, regeneration, and healing [1–3]. The stem cells should be accessible in large quantities, and the procedure of collection and harvesting of them should be non or minimally invasive, so then they can be used in regenerative medicine approaches. In addition, the differentiation of stem cells along multilineage pathways can be carried out in a reproducible manner. Then, the transplantation of them to autologous or allogeneic host is safe and effective, and their manufacturing is performed in accordance with current Good Manufacturing Practice guidelines [1, 3]. According to the origin, classification of stem cells is the following: embryonic stem cells (ES cells) [4], fetal stem cells [5], and adult (postnatal) stem cells [2, 6]. Although embryonic stem cells display enormous potential related to their pluripotency, many restrictions as well as ethical concerns are hindering their clinical applications. Facing such limitations, the need to generate an alternative source of pluripotent stem cells has emerged. The efforts succeeded in 2006, when Takahashi and Yamanaka announced the derivation of induced pluripotent stem (iPS) cells from mouse somatic cells by transduction of four defined transcription factors [7]. These adult cells reprogrammed to embryonic-like states offer a great perspective for regenerative medicine, enabling the development of patient-specific therapies [8]. There has been also an increased interest in adult stem cells as a promising tool for tissues repairing. Numerous studies have focused on bone marrow as a primary source of human adult stem cells [1, 3]. The bone marrow is considered to contain two major stem cell populations: hematopoietic (HSCs) and mesenchymal stem cells (MSCs). The latter exhibit the plastic adherent growth and extensive expansion under specific culture conditions [6, 9, 10]. However, the presence of MSCs has been also identified in other tissues and organs, such as umbilical cord blood, peripheral blood, skin, adipose tissue, skeletal muscle, gut, liver, lung, and brain [1]. In response to appropriate culture conditions, the MSCs have the ability to differentiate into mesodermal cells—osteocytes, chondrocytes, and adipocytes [6]. The capacity of mesenchymal stem cells to differentiate into other cell types of mesodermal and nonmesodermal origin remains a matter of debate [11], although differentiation into endothelial cells [12], cardiomyocytes [13], hepatocytes [14], and neural cells [15] has been reported. Such multipotential properties are not universally accepted because of the lack of globally standardized methods for their isolation, expansion, and identification, as well as the range in assays used to define terminally differentiated, functionally mature populations. Additionally, it has been described that bone marrow-derived mesenchymal stem cell cultures contribute to many tissues upon transplantation not through differentiation into mature cell types but through fusion with endogenous cells [16], making the claims for in vivo differentiation potential into other cell types controversial. Thus, it remains elusive which multipotential properties the mesenchymal stem cells really possess [11]. The endogenous roles of MSCs are the maintenance of the hematopoietic stem cell niche, organ homeostasis, wound healing, and aging. For all these reasons, MSCs are an attractive source of stem cells for therapeutic usage, and their transplantation may have a promising potential in organ repair.

In the context of clinical applications, mesenchymal stem cells originated from human adult fat depots, known as adipose-derived stem cells (ASCs), are of great importance, because of their high accessibility with minimal invasiveness and no ethical limitations. Besides the fact that mesenchymal stem cells from adipose tissue are more heterogeneous [17], they exhibit immunomodulatory properties [9, 18, 19] and differentiation ability similar to bone marrow-derived MSCs [20]. Importantly, the adipose tissue contains higher densities of adult mesenchymal stem cells, comparing to bone marrow [6]. Therefore, in this article, we have reviewed the medical literature describing the adipose tissue-derived mesenchymal stem cells as they seem to have a promising potential in regenerative medicine. Our goal was to highlight the variety of factors which may affect ASC behavior. We also noted that ASCs from distinct locations within human body may differ in their functions and characteristics. Due to the fact that the usage of ASCs in clinical applications requires the well-defined and homogenous cell population, we have tried to summarize, based on current knowledge, the molecular characterization of ASCs. We have particularly focused on trophic activity, since this feature is believed to be in a greater importance for regenerative medicine applications, rather than cell engraftment to the site of injury and their differentiation ability.

## 2. Adipose Tissue-Derived Mesenchymal Stem Cell Characteristics

### 2.1. Nomenclature

Mesenchymal stem cells isolated from adipose tissue are described by various terms which include adipose-derived stem/stromal cells (ASCs), adipose-derived adult stem (ADAS) cells, adipose-derived adult stromal cells, adipose-derived stromal cells (ADSCs), adipose stromal cells (ASC), adipose mesenchymal stem cells (AdMSCs), lipoblasts, pericytes, preadipocytes, and processed lipoaspirate (PLA) cells. Therefore, to prevent a confusion in the literature related to the use of different nomenclature, the International Fat Applied Technology Society reached a consensus to adopt the term “adipose-derived stem cells” (ASCs) to identify the isolated, plastic-adherent, multipotent cell population [1, 3]. According to these recommendations, we use the name “ASCs” in this review.

### 2.2. The Variety of Fat Depots as the Sources of Mesenchymal Stem Cells

Adipose tissue is a highly complex tissue of mesodermal origin. It comprises multiple cell types, including mature adipocytes, preadipocytes, fibroblasts, vascular smooth muscle cells, endothelial cells, resident monocytes/macrophages, and lymphocytes [21, 22]. At present, it is known that apart from energy storage function the adipose tissue is an important endocrine tissue and a source of multipotent mesenchymal stem cells.

**Figure 1:**
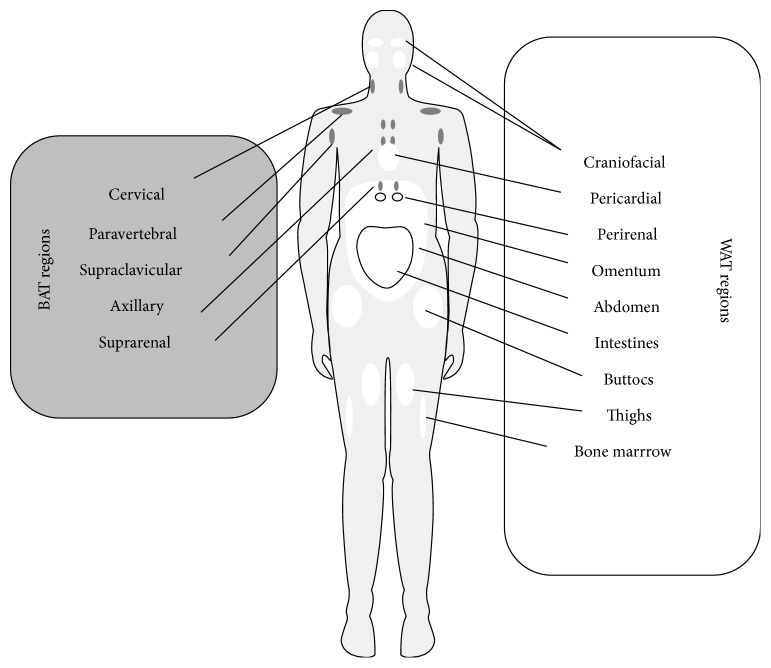
Distribution of brown (BAT) and white (WAT) adipose tissues within the human adult body [3, 23–25].

According to the developmental origin, adipose tissue can be classified into two main categories: brown and white adipose tissue, BAT and WAT, respectively [23]. Brown adipose depots are responsible for energy expenditure, whereas white adipose tissue stores energy and provides insulation. The main localizations of these two types of adipose tissue within the human body are presented in Figure 1. BAT occurs in axillary, cervical, perirenal, and periadrenal regions in fetus and newborn and is transformed to WAT during aging. Although BAT has been believed to have an insignificant function in adults, some data indicate that metabolically active brown fat can be found in the cervical, supraclavicular, axillary, paravertebral, and suprarenal regions of adult individuals [24, 25]. In turn, depots of white adipose tissue are dispersed in diverse locations such as intraabdominal and subcutaneous sites and may exhibit differentiation potential differences. For instance, Kim et al. [26] have observed the higher proliferation and adipogenic differentiation capacity in subcutaneous ASCs, compared to ASCs from intraabdominal region. The main intraabdominal WAT depots are located around the omentum, intestines, and perirenal areas, and subcutaneous depots are present in the abdomen, buttocks, and thighs. However, WAT is also located in other regions, such as retroorbital space, on the face, and extremities, supporting the eye, hand, and other critical structures. Additionally, WAT depots are found within the bone marrow, where they both occupy space no longer required for hematopoiesis and represent an energy reservoir and cytokine source for osteogenesis and hematopoiesis [3, 24]. Among various fat areas, the subcutaneous depots have been in a particular interest due to their availability, abundance, and renewability. There are two different localizations of subcutaneous fat in the abdominal region, namely superficial adipose tissue (SAT) and deep adipose tissue (DAT), and mesenchymal stem cells from these sources may be different. Di Taranto et al. [27] have noted that ASCs from subcutaneous regions displayed a slightly higher osteogenic potential, indicated by higher expression level of osteogenic marker genes, in comparison to ASCs derived from deep adipose tissue. However, adherent cells isolated from SAT and DAT showed comparable proliferation capacity and adipogenic potential. They have also found that SAT contained a higher stromal tissue compound, along with a higher proportion of CD105-positive cells, than DAT from the same harvesting site. What is more, based on the significantly higher expression levels of stemness-related transcription factors, the cells isolated from SAT were thought to exhibit increased multipotency and stemness properties [27].

Indeed, adipose tissue is promoted as an attractive stem cell source for clinical applications, due to the possibility of ASCs obtaining in significant quantities under local anesthesia using a minimally invasive procedure with no ethical concerns. As we previously mentioned, adult mesenchymal stem cells occur in higher densities in adipose tissues, rather than in the bone marrow [6]. The yield of these cells is from ~40- [18] even up to 500-fold greater in fat depots, compared to bone marrow [28, 29].

### 2.3. Factors Affecting Adipose Tissue-Derived Mesenchymal Stem Cells Characteristics

As a reservoir of adult mesenchymal stem cells, the adipose tissue is usually harvested via two different procedures: standard en bloc resection or lipoaspiration. What is important, the viability, yield, and growth characteristics of ASCs are affected by the type of harvesting procedure. Vermette et al. [30] demonstrated that cell yield obtained at the time of extraction was 1.8 times greater for lipoaspiration-derived cells, and they proliferated similarly or slightly better in culture than cells derived from resection. However, it has been shown that ultrasound-assisted liposuction resulted in a lower frequency of proliferating ASCs, as well as a longer population doubling time, compared with resection [31]. Alharbi et al. [32] compared conventional Coleman [33] versus microfat-harvesting procedure, in the context of the influence of these two fat harvesting techniques on such biological properties of ASCs as cell yield, viability, secreted growth factors concentrations or migration, and adhesion rate. The study has shown only slight differences in the yields of ASCs from abdominal subcutaneous fat tissue, obtained by these two methods. Likewise, no significant effects on the in vitro viability of lipoaspirates were found. However, the viability and migration of isolated ASCs gained from microharvested lipoaspirates were significantly higher. Furthermore, the study has revealed a significant high adherence rate of isolated ASCs from the microfat-harvesting technique onto collagen matrices. In addition, significantly higher contents of growth factors such as insulin-like growth factor (IGF) and vascular endothelial growth factor (VEGF), but not platelet-derived growth factor (PDGF) or basic fibroblast growth factor (bFGF), were observed in conventionally obtained lipoaspirates. The authors indicated the different sizes and surface/volume ratios of pieces of fatty tissue obtained using different cannula sizes as the factors responsible for the observed effects. Similarly, the study carried out by Trivisonno et al. [34] has determined the significantly higher ASCs yield in samples collected with the microcannula, compared to a standard cannula.

Summarizing, simple surgical procedure as well as easy to perform isolation and culturing protocols promotes adipose tissue to be used in clinical applications as MSC source. However, the isolation methods differ between laboratories. Mostly, the cell preparation is based on the procedure described by Rodbell [35–37]. The first step of isolation is tissue mincing, followed by enzymatic digestion with collagenase type I. The heterogeneous fraction of cells obtained immediately after collagenase digestion, which constitutes a population of adult mesenchymal stem cells and endothelial progenitor cells, is named the stromal-vascular cell fraction (SVF). After differential centrifugation, the pelleted SVF cells are placed in culture and the adherent cell population is then expanded.

Adult stem cells are influenced by many biochemical and biophysical stimuli in their in vivo microenvironment, including fluid shear stress, hydrostatic pressure, and trophic factors. It is worth to note that multiple external factors may also affect the ASC biology. Thus, it is very important to apply proper culturing conditions, such as plating density, media composition, and time of contact to plastic surface, which may influence both proliferation rate and differentiation potential of ASCs [38]. Due to the fact that ASCs exhibit surface adherence, the seeding density seems to be one of the main critical conditions affecting their biological functions. The lower seeding densities are associated with maintenance of high proliferation rate and multipotentiality. Sequential passages may influence on the quality of cultured MSCs, with progressive senescence, slowed proliferation rate, and cells progressively experiencing loss of multipotentiality [38]. Therefore, finding the correlation between seeding density and optimal cell proliferation as well as appropriate limiting of the population doublings number is useful in both laboratory studies and clinical applications because it allows the cell culturing procedure to be less time consuming, with a lower risk of cell culture contamination, and more cost effective [38, 39]. It is worth to emphasize that in terms of time of culture and functions the culture media are not equivalent and may have an impact on the behavior of the final product. Thus, the safety and efficacy of MSCs produced by distinct culture media should be carefully tested and documented [38]. It has been shown that different serum-free media based on cocktails of growth factors can maintain the main phenotypic and functional properties of cultured mesenchymal stem cells, but they require upgrading for clinical usage [40]. For example, the growth properties as well as neurotrophic and angiogenic effects of ASCs cultured in a defined xeno-free, serum-free medium were investigated by Brohlin et al. [41]. At early passage, ASCs performed better proliferation in serum-free medium, compared with standard *α*-MEM-containing fetal calf serum. However, CFUs were significantly lower in serum-free medium. ASCs cultured in serum-free medium continued to expand faster than cells grown in serum, contrary to BM-MSCs, which exhibited senescence features. In addition, stimulated ASCs and BM-MSCs expanded in serum-free medium displayed high levels of neurotrophic and angiogenic activity [41].

It has been suggested that a long-term culture with a high number of population doublings may result in undergoing of expanded MSCs to senescence and genetic instability, contributing to an increased risk of transformation or chromosomal aberrations. Indeed, there were two reports presenting spontaneous transformation and/or aneuploidy of ASCs, following long-term in vitro culture [42, 43]. However, they were retracted based on data indicating tumor cell cross-contamination artifacts. Nevertheless, testing the genetic stability of expanded mesenchymal stem cells is very relevant, both for the correct interpretation of biological outcomes and for ensuring the safety of potential stem cell therapy. It has been also hypothesized that physiological stress or in vitro culture conditions may significantly lead to the occurrence of cell or chromosomal abnormalities [44]. For instance, the enzymatic cell dissociation such as trypsinization raises more concerns in relation to abnormalities than mechanical dissociation. Thus, the culture conditions should be well defined and regularly controlled, to avoid the occurrence of karyotypic alterations [45].

It seems to be obvious that the attachment and proliferation rate are more prominent in ASCs from younger patients compared to older donors. To confirm, in the study performed by Choudhery et al. [46], aged ASCs exhibited reduced viability and proliferation when compared to cells obtained from young donors. Aged ASCs displayed increased senescent features, indicated by higher expression of senescence markers—p16 and p21 genes. These features were also associated with significantly reduced osteogenic and chondrogenic differentiation potentials in aged ASCs compared to young ASCs. It has been considered that stem cell aging is largely affected by the epigenetic modification of the genome such as DNA methylation and/or chromatin remodeling [47]. It has been suggested that DNA methylation plays a crucial role in adult stem cell aging, as DNA methylation increases with donor age in ASCs. Yan et al. [48] have found a decrease in proliferation rate as well as an impaired osteogenic differentiation potential of ASCs from aged donors, which was accompanied by a strong DNA methylation. Jurgens et al. [49] have found that adipose tissue-harvesting site may be responsible for the differences in the yield of ASCs, but no impact on total number of nucleated cells in the SVF or the ASC proliferation and differentiation capacities was observed. They indicated abdomen as a more preferable site for harvesting ASCs than the hip/thigh region. Padoin et al. [50] have compared the cell concentration of processed lipoaspirate cells in 6 commonly used donor sites for fat grafting such as the upper abdomen, lower abdomen, trochanteric region, inner thigh, knee, and flank. They have evidenced that both lower abdomen and inner thigh have higher concentrations of processed lipoaspirate cells, what points to these regions as the better donor sites of ASCs. Another factor, which may affect ASC yield, is BMI. Aust et al. [51] have shown a significant negative correlation between the number of cells obtained per milliliter of lipoaspirate with the BMI. The research group of van Harmelen et al. [52] has found that although there was an increase in the total number of fat cells and stromal cells with BMI, there was a negative correlation between BMI and number of fat cells and stromal cells per gram of adipose tissue. This decrease of the number of cells per gram of adipose tissue might be explained by the enlargement of the fat cells with increasing BMI. In addition, the chronic disease may also have an impact on ASC characteristics. For example, stem cell phenotypes may be negatively impacted by diabetes [53], and diabetic ASCs display an impaired neovascular potential in vitro [54, 55].

**Figure 2:**
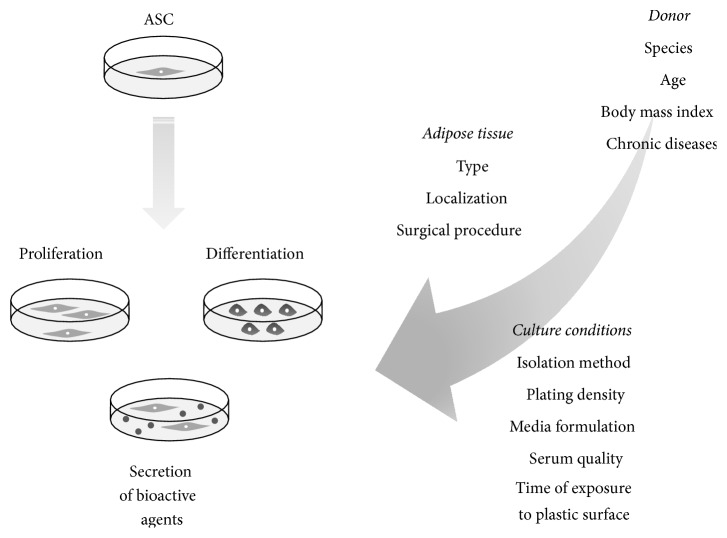
A variety of factors influencing adipose tissue-derived mesenchymal stem cell properties (proliferation capacity, differentiation potential, and trophic effects) [19, 32, 34, 38, 41–46, 48–55].

Thus, as we mentioned above, the type and localization of adipose tissue as well as surgical procedure may also have an impact on ASCs biology. The factors influencing ASC biological processes are presented in Figure 2.

### 2.4. Trophic Activity of Adipose Tissue-Derived Mesenchymal Stem Cells

The MSCs promote damaged tissue recovery by proliferating and differentiating cells, which are progeny of the engrafted cells. This feature was initially thought to be the most crucial for stem cell-based therapies' success. However, it has been reported that the implanted cells may stimulate the endogenous healing potential by their trophic activity, which attracts host progenitor cells and leads to tissue regeneration by local and invading cells [56]. Therefore, the MSC capacity to supply reparative molecules such as growth factors and cytokines to repairing tissue is believed to be in a greater importance for regenerative medicine applications, rather than MSC engraftment to the site of the lesion and their differentiation ability. It has been confirmed that ASCs are superior in secretion of bioactive factors that may stimulate cell proliferation, differentiation, and migration of various cell types such as fibroblasts, endothelial, and epithelial cells [57]. In addition, ASCs have ability to deliver protective and/or supportive factors, which may reduce apoptosis, fibrosis, and inflammation [58], in significantly higher quantities and numbers than MSCs from bone marrow [59]. For instance, Hsiao et al. [60] have found that of all examined MSC populations, ASCs represented the most attractive cell type for promoting angiogenesis in tissue engineering applications, expressing at higher levels some paracrine factors such as insulin-like growth factor-1 (IGF-1), vascular endothelial growth factor-D (VEGF-D), and interleukin-8 (IL-8). ASCs showed a significantly greater angiogenic potential compared with BM-MSCs in a study performed by Kim et al. [61]. Moreover, the factors released by MSCs may suppress the local immune system, by modulating T and B cells and inducing the expression of anti-inflammatory factors, such as interleukin-10 (IL-10), IL-1 receptor antagonist (IL-1Ra), or prostaglandin E2 (PGE2) [62–64].

**Figure 3:**
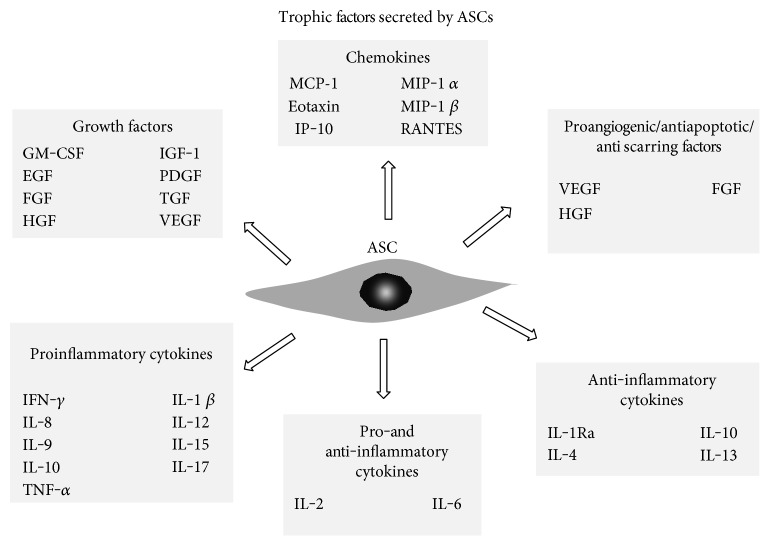
Different types of trophic factors released by adipose tissue-derived mesenchymal stem cells [27, 65–69, 76, 99].

The ASCs produce a larger number of growth factors than bone marrow MSCs, which include granulocyte-macrophage colony stimulating factor (GM-CSF) [58, 59], granulocyte-colony stimulating factor (G-CSF), IL-1Ra, IL-8, and HGF [59]. Additionally, they produce a large number of other factors, such as transforming growth factor (TGF) [58], vascular endothelial growth factor (VEGF) [59, 65], platelet-derived growth factor (PDGF) [66], fibroblast growth factor (FGF) [67], hepatocyte growth factor (HGF) [68], and members of epidermal growth factor family (EGF) [69] (Figure 3()). Secretion of angiogenic and antiapoptotic growth factors at bioactive levels makes the subcutaneous ASCs a novel source for cardiovascular therapies. In fact, some clinical trials using ASCs in cardiac therapy have been succeeded [70]. The first study, A Randomized Clinical Trial of AdiPOse-derived Stem ceLLs in the Treatment of Patients With ST-elevation myOcardial Infarction—the APOLLO Trial (NCT00442806), investigated the safety and feasibility of intracoronary infusion of autologous adipose-derived stem and regenerative cells in acute myocardial infarction patients after successful revascularization. The data reported at the 7th International Symposium on Stem Cell Therapy and Cardiovascular Innovation showed the improvement in the left ventricular ejection fraction (LVEF), reduction in infarct size, and improvement in myocardial perfusion [71]. The ADVANCE Study (NCT01216995) further evaluated the efficacy of this approach defined as reduction in infarct size at 6 months. Another clinical trial targeted to patients with chronic ischemic heart disease, MyStromalCell Trial (NCT01449032), completed in 2014, was evaluating the efficacy and safety of intramyocardial delivery of VEGF-A_165_-stimulated autologous adipose tissue-derived MSCs to improve myocardial perfusion and exercise capacity and reduce symptoms. The outcomes of another trial, the PRECISE trial (https://www.clinicaltrials.gov/NCT00426868), suggested that adipose-derived regenerative cells may preserve ventricular function, myocardial perfusion, and exercise capacity in patients suffering from chronic ischemic cardiomyopathy [72].

In turn, Sawada et al. [73] have reported the importance of the trophic effects of adipose tissue-derived multilineage progenitor cells (ADMPCs) in the periodontal tissue regeneration. The growth factors released by ADMPCs included HGF, VEGF, and insulin-like growth factor binding protein 6 (IGFBP6). Among those, HGF participates in the proliferation and migration of vascular endothelial cells. The second factor, VEGF, plays critical roles in blood vessel formation through endothelial cell proliferation and migration. Furthermore, the expression of VEGF and its receptors has been confirmed in periodontal ligament cells (PDLs) and gingival fibroblasts in periodontal tissues. It is also known that VEGF promotes proliferation and migration of human periodontal ligament cells (HPDLs) and their differentiation into osteoblasts [74, 75]. For these reasons, HGF and VEGF secreted by ADMPCs are suggested to contribute to periodontal tissue regeneration in a non-cell-autonomous manner. IGFBP6, the most highly presented in ADMPC-conditioned medium, was shown to stimulate the differentiation of HPDLs to mineralized tissue-forming cells [73].

In addition to growth factors, the ASCs produce a variety of other molecules, such as cytokines and chemokines. For example, proinflammatory cytokines secreted by ASCs include IFN-*γ*, IL-1*β*, IL-7, IL-8, IL-9, IL-12, IL-15, IL-17, and TNF-*α*, anti-inflammatory cytokines—IL-1Ra, IL-4, IL-10, and IL-13 [59]. Moreover, the ASCs produce IL-2 and IL-6 that are considered to display both pro- and anti-inflammatory effects under different conditions. In turn, the ASCs secrete some chemokines such as MCP-1, eotaxin, IP-10, MIP-1*α*, MIP-1*β*, and RANTES [59, 76] (Figure 3). However, it has been found that the cytokine secretion profiles vary between ASCs from diverse locations. For instance, Naftali-Shani et al. [77] have demonstrated that human mesenchymal stromal cells isolated from patients with ischemic heart disease from the right atrium and epicardial fat secreted the highest amounts of trophic and proangiogenic factors, namely HGF, basic FGF, and PDGF, in comparison to pericardial and subcutaneous fat depots. Moreover, the immunomodulatory cytokines, such as TNF-*α*, tissue inhibitors of metalloproteinase 2, and IL-13, were produced in the highest amounts also by MSCs from epicardial fat and the right atrium of those patients. However, despite the higher levels of trophic and angiogenic factor secretion, MSCs from epicardial fat and the right atrium exerted the inferior effect on cardiac remodeling and function, as evidenced by the highest inflammation score in the infarcted heart, compared to subcutaneous fat MSCs [77]. Furthermore, whereas the accumulation of visceral fat is associated with increased prevalence of insulin resistance, metabolic syndrome, and related cardiovascular complications [78], factors secreted by subcutaneous fat MSCs may mediate the antiatherogenic effects of subcutaneous fat [79]. In turn, the experiments performed by Mazurek et al. [80] have shown that epicardial adipose tissue is a source of several inflammatory mediators in high-risk cardiac patients. In case of suffering from coronary artery disease (CAD), the epicardial adipose tissue displayed significantly higher levels of chemokine (MCP-1) and several inflammatory cytokines (IL-1*β*, IL-6, IL-6sR, and TNF-*α*), comparing to subcutaneous adipose tissue. The inflammatory mediators present in epicardial adipose tissue could lead to amplification of vascular inflammation, plaque instability via apoptosis (TNF-*α*), and neovascularization (MCP-1) [80].

Particular attention should be given to exosomes, since it is known that they contribute to the paracrine effects of MSCs [81]. Exosomes are small, intraluminal vesicles of multivesicular bodies released when they fuse with the plasma membrane [82]. It has been suggested that these vesicles are secreted by a variety of cell types and can function as intercellular transmitters of mRNA, microRNA, and proteins [83]. They are thought to mimic the roles played by mesenchymal stem cells from which they originate [84, 85]. The importance of ASC-secreted exosomes in promoting tissue repair has been reported. For instance, Hu et al. [86] have indicated that exosomes of adipose tissue may promote beneficial effect for soft tissue wound healing. They have found that exosomes released by ASCs could be internalized by fibroblasts to stimulate cell migration, proliferation capacity, and collagen synthesis in a dose-dependent manner. In turn, the exosomes from ASCs have been considered as a therapeutic agent for the treatment of inflammation-related diseases by Blazquez et al. [87], due to their inhibitory effect in the differentiation and activation of T cells as well as a reduced T cell proliferation and IFN-*γ* release on in vitro stimulated cells. Furthermore, Katsuda et al. [88] have demonstrated that exosomes secreted by ASCs contained enzymatically active neprilysin (neutral endopeptidase: NEP or CD10), involved in the degradation of *β*-amyloid peptide (A*β*) whose accumulation in the brain plays a critical role in Alzheimer's disease pathogenesis. Ascending evidence have suggested that exosomes might be the main components of paracrine factors, thus, they may represent a novel therapeutic tool in regenerative approaches. However, prior to clinical applications, the oncogenic risks that may be associated with MSC-derived exosomes should be overcome [84].

### 2.5. Surface Markers of Adipose Tissue-Derived Mesenchymal Stem Cells

Depending on the source of origin, the MSCs show differences in gene expression, surface epitopes, clonogenicity, ability to differentiate, and therapeutic potential. Surface marker profile of MSCs varies between species. Furthermore, these cells express different molecules according to the isolation and culture procedure [89, 90]. Expression of surface markers can be also conditioned by factors produced by the accessory cells in the initial culture period. In addition, the expression of some molecules may vary in vitro and in vivo [91]. In tissue, MSCs reside at various stages of differentiation. Therefore, in culture conditions, these cells exhibit heterogeneity in marker expression, renewal capacity, and differentiation potential [92]. The MSCs have been identified by a set of nonspecific surface antigens, but there are no definitive surface markers for the exclusive isolation of these cells. Therefore, prior to clinical applications, the challenges concerning the isolation, identification, and purification of stem cells must be overcome. The defining of MSC specific marker proteins, to use the well-identified and homogeneous cell population, is pivotal.

**Figure 4:**
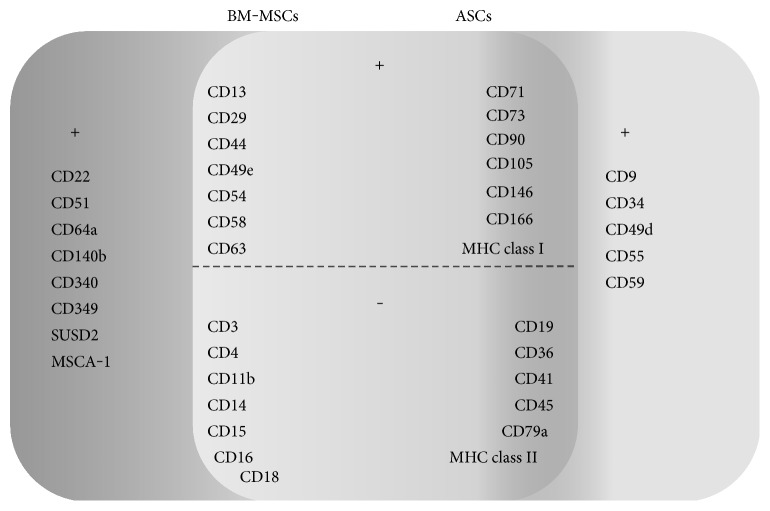
Comparison of surface marker profile of mesenchymal stem cells from two distinct origins, bone marrow (BM-MSCs) and adipose tissue (ASCs) [1–3, 36, 93–98].

Phenotypically, MSCs have been described as CD29, CD44, CD90, and CD105 positive and negative for hematopoietic lineage markers and HLA-DR [18]. There are some attempts to well define the surface markers specific to ASCs, distinguishing them from BM-MSCs (Figure 4()). It has been demonstrated that ASCs expressed classical MSC markers such as CD29-beta-1 integrin, involved in therapeutic angiogenesis, CD44-hyaluronate receptor, which is crucial in the development of neoextracellular matrix and is involved in many pathologic and physiologic phenomena, and were absent for HLA-DR and c-kit expression. Other surface molecules expressed by both ASCs and BM-MSCs include CD13, CD49e, CD54, CD63, and CD146 [17, 19] (Figure 4()). Furthermore, cultured adipose tissue-derived stem cells display the expression of classical bone marrow MSC surface markers, such as CD73, CD90, CD105, and the lack of expression of haematopoietic (CD14, CD45) and endothelial markers (CD31) [9]. However, it has been revealed by Mitchell et al. [93] that the latter was expressed on SVF cells and did not change significantly with serial passage. What is more, they observed that other endothelial cell-associated markers, such as CD144 (VE-cadherin), VEGFR-2, and von Willebrand factor, were expressed on crude SVF cells. The levels of these molecules did not change much through the culture period [93]. Importantly, no expression of hematopoietic markers such as CD3, CD11b, CD14, CD15, CD16, CD18, and CD41 was observed [21]. Other molecules not expressed by both ASCs and BM-MSCs include CD4, CD19, CD36, and CD79a [9, 21]. The studies performed by Mitchell et al. [93] have shown that the immunophenotype of ASCs has progressively changed with adherence and passage. The levels of stromal cell-associated markers, including CD13, CD29, CD44, CD63, CD73, CD90, and CD166, were initially low on SVF cells and enhanced significantly with serial passage. The ALDH, used to identify the hematopoietic stem cells, was present at a high level (more than 70%) to passage 4 [93]. In addition, there is a report indicating the expression of CD71 and MHC class I by both ASCs and BM-MSCs [94]. It is worth to note that the data referring to the surface molecule characteristic for ASCs are inconsistent. For instance, CD106 has been described in the literature both as expressed [21] and not expressed [31] by adipose-derived stem cells. It has been demonstrated that native ASCs belong to the CD34+ cell fraction of the adipose tissue SVF [95]. However, other groups also suggest the existence of mesenchymal stem cell population derived from CD34 cells within adipose tissue [96]. Mitchell et al. [93] have observed that about 60% of the initial SVF cells showed expression of CD34, but the percentage of positive cells reduced in successive passage. Likewise, studies performed by Maumus et al. [95] have shown that CD34 expression decreased during the culture period and was negatively correlated with cell proliferation rate. Moreover, some data have revealed that ASCs are CD166 (activated lymphocyte common adhesion molecule) and STRO-1 positive [20]. However, it has been found that the latter, which is the best known MSC marker, is expressed at higher levels by ASCs cultured beyond the first passage or in case of inducing them to differentiate into endothelial cells. This indicates that STRO-1 is intrinsically an endothelial antigen, and its expression in MSC might be inducible [97]. Interestingly, ASCs in their native microenvironment were negative for CD140b and NG2 pericyte markers but the expression of them was induced by culture process [95].

There are some attempts to distinguish the population of bone marrow mesenchymal stem cells, from those of adipose tissue origin. Busser et al. [98] have demonstrated the lack of expression of SUSD2 and MSCA-1 by mesenchymal stem cells from adipose tissue in situ, in contrast to MSCs from bone marrow. What is more, BM-MSCs have been shown to be positive for CD22, CD51, CD64a, CD340, and CD349, comparing to ASCs [94]. In turn, the researchers have observed that other marker, CD271, allows to define adipose tissue cell subsets with particular abilities, but only in lipoaspiration samples and not in abdominoplasty samples [98]. It has been also reported that CD58 and CD117 were expressed by both bone marrow and adipose tissue mesenchymal stem cell populations [94]. On the other hand, some reports exert that all types of MSCs are characterized by lack of CD117 expression [9]. In contrast to cells derived from bone marrow, the ASCs exhibited the expression of CD49d. This receptor forms a heterodimer with CD29 to create VLA-4 (very late activation antigen-4), the main cognate ligand for vascular cell adhesion molecule-1 [VCAM-1]–CD106 ligand [94]. Moreover, it might be suggested that CD9, CD49d, CD55, and CD59 are molecules distinguishing mesenchymal stem cells derived from adipose tissue and bone marrow [20, 94]. In comparison to MSCs from bone marrow, the ASCs display high levels of CD54 (intercellular adhesion molecule-1 [ICAM-1]) [20]. This protein, a member of the immunoglobulin superfamily, can be upregulated in response to some inflammatory mediators and cytokines.

## 3. Conclusion

Over the last few years, there has been an increasing demand on therapeutic usage of stem cells in regenerative medicine. Mesenchymal stem cells can be easily obtained from patient's own tissues, isolated ex vivo, expanded, and transplanted back into the patient as an autologous transplant. Additionally, MSCs are real candidates for cellular therapy in allogeneic approaches, because of their immunosuppressive properties. Adipose tissue may serve as an abundant and accessible source of adult stem cells that can be used as an alternative to bone marrow mesenchymal stem cells in regenerative medical therapies. The ASCs are one of the cell populations found in stromal vascular fraction of adipose tissue. The adipose tissue-derived mesenchymal stem cells are capable of differentiating along multiple pathways. In addition, isolation and culture procedures are easy to perform.

Noteworthy, ASC property of secreting or making other cells in the vicinity to secrete functionally active agents is particular. It is believed that this feature is especially important for the success of tissue regeneration and repair after injury. ASCs are known to provide trophic immunosuppressive and anti-inflammatory effects through production of a variety of growth factors, cytokines, and chemokines (Figure 3()). However, there are some reports indicating that secretion of these bioactive factors may vary between different fat regions [77, 80]. Moreover, the differences in trophic activity of ASCs may even occur within the same adipose tissue region, such as in the superficial and deep adipose tissues which both belong to abdominal region of subcutaneous fat [27]. It has been also observed that the production of trophic agents, namely G-CSF, GM-CSF, IL-6, IL-7, IL-8, IL-15, HGF, NGF, VEGF, IP-10, eotaxin, and IL-1Ra, reveals donor to donor variations [59]. This means that microenvironmental cues, like stimuli from growth factors, have a significant impact on stem cell “behavior”. Thus, the cells forming the niche are no less important than stem cells that occupy and respond to this microenvironment.

Mesenchymal stem cells have been determined by a set of nonspecific surface proteins (Figure 4). However, for clinical applications, the identification of surface markers for the exclusive isolation of these cells is crucial. Importantly, to precisely define the MSC population, a combination of surface antigens and gene expression parameters is required; therefore, a single marker might not be sufficient. It has been indicated that some conditions may cause differences in the surface molecules expression. For instance, diverse collection and processing methods, quality of serum, or donor specificity may lead to differences in the composition and characteristics of MSC populations. Furthermore, surface marker expression profile is also known to be dependent on species. Although ASCs express surface markers similar to BM-MSCs, some variations occur. Such differences may also be related to the age of donors.

The literature reports concerning the immunophenotype of ASCs remain inconsistent. Primarily, it is worth noting that the expression pattern of markers analyzed in vitro does not always reflect the characteristics in vivo. For instance, while during culture period ASCs expressed CD140b, a cell surface tyrosine kinase receptor for members of the PDGF family, in vivo the lack of expression was observed [95]. Therefore, the expression of some molecules might be inducible. As it was mentioned above, during the culture period, the alterations of surface molecule expression level may occur. Some stromal cell-associated markers are initially expressed on a low level by SVF cells, whereas with serial passage, their increase is reported. On the other hand, it has been shown that a CD34, predominantly regarded as a marker of hematopoietic stem and progenitor cells, is expressed by most of the SVF cells but its level decreases during culture [93, 95]. Some endothelial markers, such as CD31, are expressed by crude SVF cells but not by cultured ASCs. It might be due to heterogeneity of SVF population, which besides the mesenchymal stem cells consists of endothelial progenitor cells. Taken together, a comprehensive and comparative analysis with other types of stem cell preparations, as well as a variety of terminally differentiated cell types, is necessary to specify a subset of reliable molecular markers.

To conclude, the unique properties of ASCs, such as self-renewal, differentiation into specialized tissues and organs, and secretion of trophic factors, in combination with supplying them to repairing tissue, make this type of mesenchymal stem cells a great tool for tissue engineering applications. Nevertheless, multiple aspects can influence adipose-derived stem cell properties and, thereby, affect the success of healing. The developmental fate of a stem cell depends on a general predetermined potential as well as on microenvironmental signals. This interaction of stem cells with their physiological environment deserves a particular attention, in the context of stem cell-based therapy development.

## Abbreviations

ALDH: Aldehyde dehydrogenase
AT: Adipose tissue
CD: Cluster of differentiation
CFU: Colony-forming unit
HLA: Human leukocyte antigens
IFN-*γ*: Interferon gamma
IGF-1: Insulin-like growth factor 1
IL: Interleukin
IP-10: IFN-gamma-inducible protein 10
MCP-1: Monocyte chemoattractant protein-1
MIP: Macrophage inflammatory proteins
MSCA-1: Mesenchymal stromal cell antigen-1
Oct-4: Octamer-binding transcription factor 4
RANTES: Regulated on activation, normal T cell expressed and secreted
SOX2: (Sex determining region *Y*)-box 2
SUSD2: Sushi domain containing 2
TNF-*α*: Tumor necrosis factor alpha.
